# Randomized comparison of effects of two different remifentanil dose on surgical conditions during endoscopic sinus surgery

**DOI:** 10.1186/s12871-023-02253-3

**Published:** 2023-08-29

**Authors:** JinHyeok Jeong, ChanWoo Park, YoungJoon Yoon, DoJae Lee, SangYun Cho

**Affiliations:** 1 Department of Otorhinolaryngology, Hanyang University Guri Hospital, Guri-Si, Gyeonggi-Do, Republic of Korea; 2 Department of Anesthesiology and Pain Medicine, Hanyang University Guri Hospital, 249-1, Gyomun-Dong, Guri-Si, Gyeonggi-Do, 471-701 Republic of Korea

**Keywords:** Endoscopic sinus surgery, Remifentanil, Surgical field condition

## Abstract

**Background:**

The combination of propofol and remifentanil results in better surgical field conditions during endoscopic sinus surgery than inhalation anesthesia. This study compared surgical field conditions between two groups receiving low or high concentration of remifentanil and hemodynamic variables using non-invasive cardiac monitoring.

**Methods:**

Fifty-four patients between ASA I or II, were randomly assigned to either the high-concentration remifentanil group (HR), effect-site concentration of 8 ng/mL or the low-concentration remifentanil group(LR), effect-site concentration of 4 ng/mL. Surgical condition was evaluated using the Boezaart Surgical Field Grading Scale presented by Boezaart. Cardiac output was measured using non-invasive cardiac monitoring (CSN-1901).

**Results:**

In terms of surgical conditions, the HR group showed significantly lower values than the LR group (*p* = 0.021) at 90 min after the start of surgery. Heart rate was significantly lower in the HR group than the LR group at 30, 60, and 90 min after the start of surgery (30 min; *p* = 0.005, 60 min; *p* = 0.002, 90 min; *p* = 0.001). There was a statistically significant decrease of cardiac output in the HR group compared to the LR group immediately after endotracheal intubation and at 30, 60, and 90 min after the start of surgery (Base; *P* = 0.222, Intubation; *P* = 0.016, 30 min; *p* = 0.014, 60 min; *P* = 0.012, 90 min; *P* = 0.008). However, in the case of stroke volume, there was no significant difference between the two groups in all measurements.

**Conclusion:**

When comparing the HR group and the LR group, the surgical condition was improved at 90 min after the start of surgery. MAP was lower in the HR group and this was a result of reduction in cardiac output primarily attributed to the decrease in heart rate rather than a decrease in stroke volume.

**Trial registration:**

Clinical Trial Registry of the Republic of Korea (KCT0006453).

## Introduction

Endoscopic sinus surgery is a widely utilized treatment for sinusitis, often performed under general anesthesia [1]. However, a significant challenge in these procedures is the increased bleeding that occurs, leading to reduced visibility and difficulties in manipulating the endoscope [2]. This can hinder the surgeon’s ability to effectively treat the underlying sinusitis.

Reducing bleeding during endoscopic sinus surgery is of paramount importance as it directly impacts the surgical outcome and patient safety. Various approaches have been investigated to mitigate this issue, and one promising method involves the use of total intravenous anesthesia (TIVA) with a combination of the intravenous anesthetic, propofol and the opioid, remifentanil [3]. Despite the potential benefits of TIVA, the optimal concentration of remifentanil for achieving improved surgical conditions also remains unclear. Determining the most effective remifentanil concentration is crucial for optimizing surgical outcomes and patient comfort. The specific concentration of remifentanil that provides optimal surgical conditions while maintaining hemodynamic stability is an area that requires further investigation.

Therefore, the primary objective of this study is to compare the effects of high-concentration (effect site concentration of 8 ng/ml) remifentanil and low-concentration (effect site concentration of 4 ng/ml) remifentanil on surgical conditions during endoscopic sinus surgery. The dosage of remifentanil was determined based on references from two papers, which have established a safe dosage for patients [4, 5]. While assessing the impact of remifentanil concentration on bleeding control and surgical visualization, we also aim to explore hemodynamic variables such as cardiac output, stroke volume, heart rate, and mean arterial pressure to evaluate the stability and safety of each remifentanil concentration using non-invasive cardiac monitoring.

## Methods

### Study subjects

The subjects were 54 patients between the ages of 18 and 65 years who were rated as American Society of Anesthesiology Classification I or II. Exclusion criteria were: 1) patients with allergies to eggs or soybean oil; 2) patients with a history of drug abuse; and 3) patients receiving other medical treatment, e.g., for hypertension, diabetes, or other conditions. After obtaining approval from the hospital’s institutional review board (Hanyang University Guri Hospital Institutional Review Board, GURI 2021-03-041-006) and registering with the clinical research information service centre (KCT0006453, Date of registration: 17/08/2021, Principal Investigator: SY Cho), this study was performed in accordance with the relevant guidelines and regulations. This study was in accordance with the declaration of Helsinki. Written informed consent was obtained from the patients who voluntarily agreed to participate in the study.

### Research methods

The study was based on a randomized trial and the subjects were randomly classified into computer-generated numbers. Instructed nurses who did not know the study details randomly assigned the subjects by selecting high concentration and low concentration from a sealed envelope just before surgery. The allocation ratio was 1:1 for each group. After the patient arrived at the operating room, electrocardiogram, pulse oxygen saturation**,** non-invasive blood pressure, and end-tidal carbon dioxide pressure were measured. Depth of anesthesia was measured using a bispectral index (BIS) monitor (A-2000TM, version 3.3, Aspect Medical Systems Inc., Newton, MA, USA). After administering 100% oxygen for three min to the patient, 40 mg of 1% lidocaine was administered intravenously. Using a target concentration-controlled injector (Orchestra, Fresenius-Vial, Brezins, France), propofol was administered at an effect-site concentration of 6.0 µg/ml using a Schnider pharmacokinetic model. Remifentanil was administered at an effect-site concentration of 2.0 - 4.0 ng/ml using the Minto pharmacokinetic model. During administration of propofol and remifentanil, the patient received the instruction "open your eyes" every 10 s. If the patient could not open their eyes, the muscle relaxant rocuronium was injected at a dose of 0.6 mg/kg. When the BIS level dropped below 60 and the train of four (TOF) on the nerve stimulator reached zero, endotracheal intubation was performed.

Administration of remifentanil during anesthesia maintenance was performed by a target-controlled infusion (TCI) device with an effective site concentration of 8 ng/ml in the high-concentration group and of 4 ng/ml in the low-concentration group. The TCI device was prepared in advance by an instructed nurse, and the fixed concentration of drug was sealed. The anesthesiologist who administered the anesthesia was, therefore, blinded to the concentration of remifentanil used. The effect-site concentration of propofol was adjusted by 0.2 µg/ml from the initial concentration to maintain a BIS value of 40–60. Ephedrine was used 5-10 mg intravenously only when MAP falls below 60 mm Hg.

At the end of the operation, propofol and remifentanil were discontinued; pyridostigmine 0.2 mg/kg and glycopyrrolate 0.008 mg/kg were injected intravenously to reverse the muscle relaxant effects; and extubation was performed under the influence of a nerve stimulator.

Baseline systolic arterial pressure (SAP), diastolic arterial pressure (DAP), mean arterial pressure (MAP), heart rate (HR), BIS, stroke volume (SV), and cardiac output (CO) levels were measured immediately after arrival at the operating room. The variables also were measured immediately after endotracheal intubation and at 30, 60, and 90 min after the start of surgery, for observation and comparison. Stroke volume and cardiac output were measured using non-invasive cardiac monitoring, for which the CSN-1901 (Nihon Kohden, Tokyo, Japan) was used. Evaluation of the surgical condition was conducted by a single operator using the Boezaart surgical field grading scale (Table 1) [6] and was recorded at 30, 60, and 90 min after the start of surgery. Although the type of surgery was the same, the pre-existing conditions of the patient varied slightly.

**Table 1:** Surgical grading scoring system designed specifically for use in endoscopic sinus surgery

Grade	Assessment
0	No bleeding (cadaveric conditions)
1	Slight bleeding – no suction required
2	Slight bleeding – occasional suction required
3	Slight bleeding – frequent suctioning requied; bleeding threatens surgical field a few seconds after suction is removed
4	Moderate bleeding – frequent suction ing required and bleeding threatens surgical field directly after suction is removed
5	Severe bleeding – constant suctioning requied; bleeding appears faster than can be removed by suction; surgical field severely threatened and surgery usually not possible

The total doses of propofol and remifentanil administered during anesthesia were recorded.

After 30 min and within 24 h of recovery room arrival, the Visual Analog Scale (VAS) and Postoperative Nausea and Vomiting (PONV) score were measured. The PONV score was divided into 4 categories, 0 = none, 1 = nausea, 2 = retching, and 3 = vomiting. Ramosetron was given to the patients who experienced PONV.

### Sample size calculation

We used the mean and standard deviation from pilot study (mean and standard deviation: 2.3 and 0.57). In a two-tailed analysis where α = 0.05 and β = 80%, 24.1 patients were required to obtain a 20% difference of the mean between the experimental and control groups. A total of 54 patients were required to maintain acceptable statistical power, assuming a 10% dropout rate. Sample Size Software, 3rd edition from Sample Size Tables for Clinical studies by David Machin, Michael Campbell, Say-Beng Tan et al. was used for calculation.

### Statistics

The Statistical Package for Social Sciences 20.0 for Windows (SPSS Inc., IL, USA) was used as a statistical program. The Mann-Whitney rank sum test was performed to compare numerical data between the two groups, and the χ2 test and Fisher’s exact test were used for non-parametric data. Kruskal-Wallis one-way analysis of variance with a Dunn multiple comparison test was used to assess haemodynamic change. The statistical significance level was set at *p* = 0.05.

## Results

**Fig. 1:**
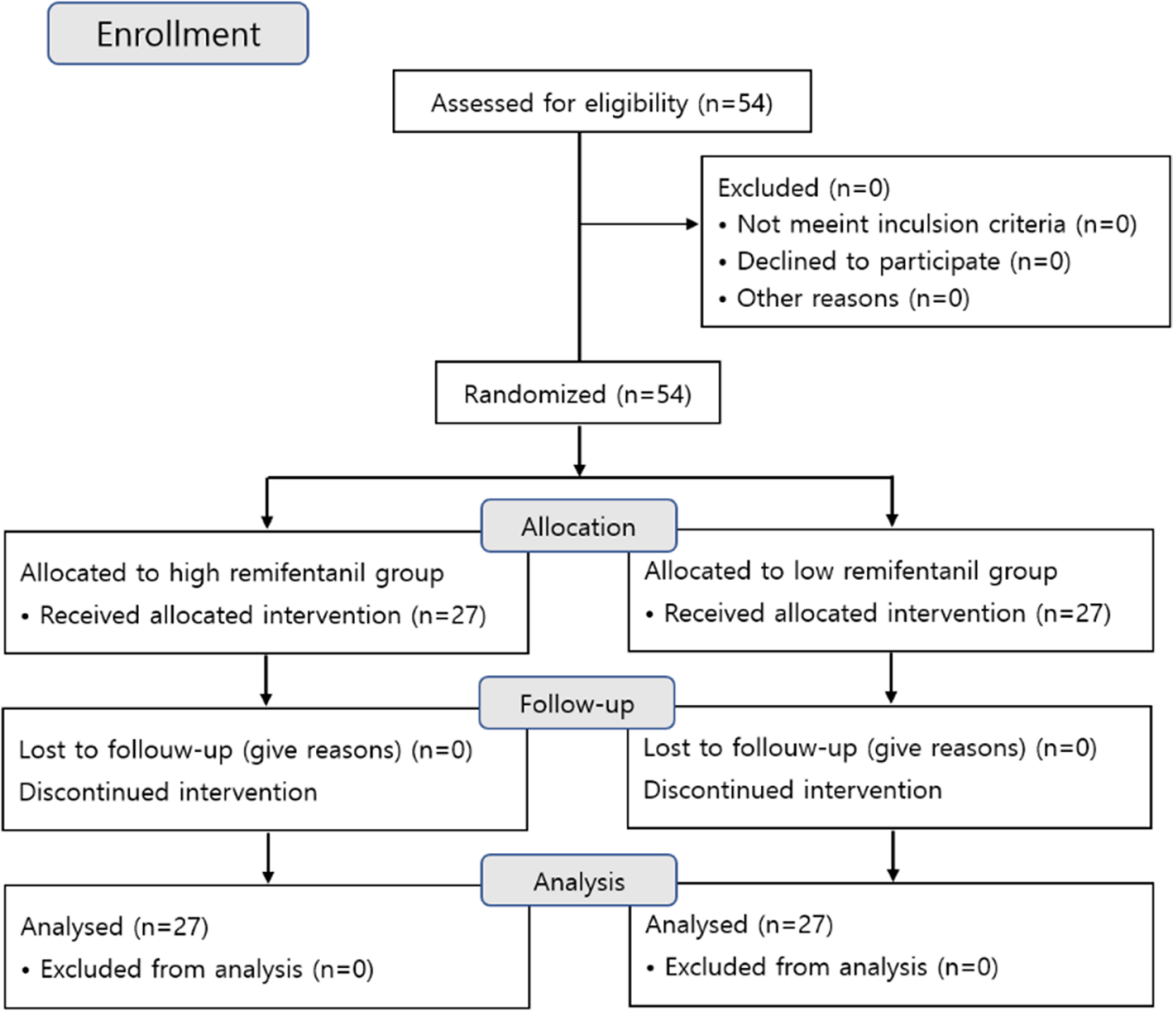
Subject flow diagram

All 54 patients who participated in the study met the inclusion criteria, and none refused to participate. These patients were randomly assigned to a high-concentration or low-concentration group, with 27 patients in each group (Fig. 1).

Subject data and clinical characteristics are presented in Table 2. There was no significant difference between the two groups in terms of mean age, gender, height, weight, body mass index, ASA score, and operation and anesthesia times.

**Table 2:** Demographic data and clinical characteristics of patients

	**Group LR (*n* = 27)**	**Group HR (*n* = 27)**
**Age (yr)**	44 ± 12.0	47.1 ± 13.0
**Gender (M/F)**	9/18	9/18
**Height (cm)**	167.1 ± 8.8	167.6 ± 9.0
**Weight (kg)**	69.7 ± 13.0	69.1 ± 14.3
**Body mass index (kg/m**^**2**^**)**	25.0 ± 4.1	24.5 ± 3.4
**ASA (I/II)**	10/17	11/16
**Operation time (min)**	108.3 ± 30.0	102.8 ± 22.8
**Anesthesia time (min)**	138.3 ± 28.2	135.7 ± 22.0

Values are number or mean ± SD

*Group LR* Low remifentanil group, *Group HR* High remifentanil group, *ASA* American Society of Anesthesiologists physical status

Hemodynamic variables between the two groups were measured through non-invasive cardiac monitoring (Table 3). For cardiac output, there was a statistically significant decrease in the high concentration group compared to the lower concentration group immediately after endotracheal intubation and at 30, 60, and 90 min after the start of surgery (cardiac output, base; *p* = 0.222, intubation; *p* = 0.016, 30 min; *p* = 0.014, 60 min; *p* = 0.012, 90 min; *p* = 0.008). However, for stroke volume, there was no significant difference between the two groups in all measurements (stroke volume, base; *p* = 0.482, intubation; *p* = 0.461, 30 min; *p* = 0.258, 60 min; *p* = 0.263, 90 min; *p* = 0.603).

**Table 3:** Haemodynamic parameters

	**Group LR (*n* = 27)**	**Group HR (*n* = 27)**
Cardiac output, Base	7.20 ± 1.45	6.31 ± 2.34
Cardiac output, Intubation	7.34 ± 1.32	5.87 ± 1.81^*^
Cardiac output, 30	6.32 ± 1.28	5.00 ± 1.53^*^
Cardiac output, 60	6.45 ± 1.47	4.98 ± 1.56^*^
Cardiac output, 90	6.83 ± 1.37	5.18 ± 1.83^*^
Stroke volume, Base	92.4 ± 14.4	87.8 ± 20.7
Stroke volume, Intubation	88.2 ± 14.5	83.9 ± 17.0
Stroke volume, 30	89.3 ± 16.1	82.5 ± 16.9
Stroke volume, 60	88.5 ± 16.4	81.4 ± 18.1
Stroke volume, 90	88.1 ± 14.9	84.8 ± 19.8

The values are measured by non-invasive cardiac monitoring with CSN-1901. Base: preoperative control values; Intubation: immediately after intubation; 30, 60, and 90: 30 min, 60 min, and 90 min, respectively, after beginning the operation

^*^p < 0.05 compared to Group LR

**Fig. 2:**
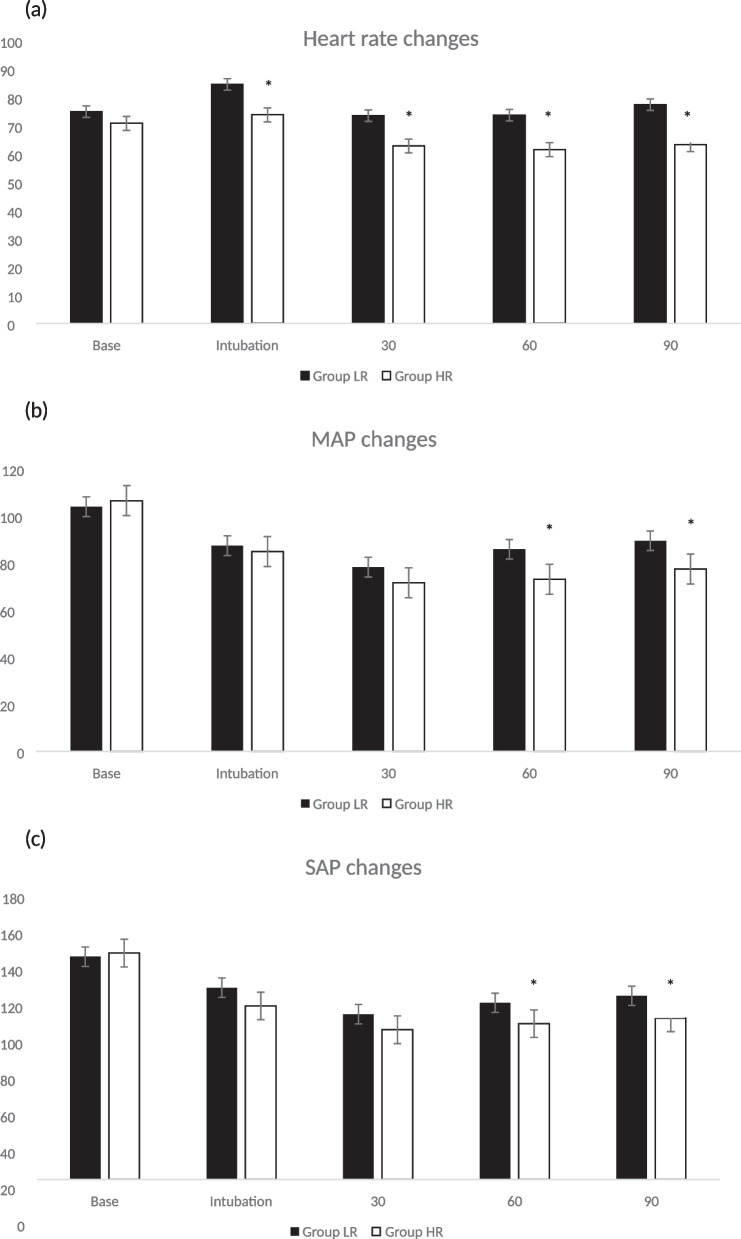
**a** Changes of heart rate in groups LR and HR. Base: preoperative control values; Intubation: right after intubation; HR 30, 60, and 90: 30, 60, and 90 respective min after beginning the operation. *p < 0.05 compared to Group LR. **b** Changes of mean arterial pressure in groups LR and HR. Base: preoperative control values; Intubation: right after intubation; HR 30, 60, and 90: 30, 60, and 90 respective min after beginning the operation. *p < 0.05 compared to Group LR. **c** Changes of systolic arterial pressure in groups LR and HR. Base: preoperative control values; Intubation: right after intubation; HR 30, 60, and 90: 30, 60, and 90 respective min after beginning the operation. *p < 0.05 compared to Group LR

The changes in heart rate between the two groups (Fig. 2a) were significantly lower in the high-concentration group compared to the low-concentration group immediately after endotracheal intubation and at 30, 60, and 90 min after the start of surgery (heart rate changes, base; *P* = 0.412, intubation; *p* = 0.033, 30 min; *p* = 0.005, 60 min; *p* = 0.002, 90 min; *p* = 0.001). Mean arterial pressure (Fig. 2b) and systolic blood pressure (Fig. 2c) decreased in the high-concentration group compared to the low-concentration group immediately after endotracheal intubation and at 30 min after the start of surgery; however, these differences were not statistically significant. At 60 and 90 min after the start of surgery, the high-concentration group showed statistically lower MAP and SBP than the low-concentration group (mean arterial pressure, base; *p* = 0.692, intubation; *p* = 0.765, 30 min; *p* = 0.121, 60 min; *p* = 0.006, 90 min; *p* = 0.025, systolic arterial pressure, base; *p* = 0.786, intubation; *p* = 0.144, 30 min; *p* = 0.081, 60 min; *p* = 0.039, 90 min; *p* = 0.019).

Surgical conditions (Table 4) did not show significant differences between the two groups at 30 and 60 min after the start of surgery. However, at 90 min after the start of surgery, the high-concentration group showed a significantly lower score than the low-concentration group (surgical score, 30 min; *p* = 0.315, 60 min; *p* = 0.269, 90 min; *p* = 0.021). There was no significant difference between the two groups in terms of postoperative pain, nausea, and vomiting (VAS score, *p* = 0.991, PONV score, *p* = 0.077).

**Table 4:** Changes of surgical scores and other parameters during endoscopic sinus surgery

	**Group LR (*n* = 27)**	**Group HR (*n* = 27)**
Surgical score, 30	2.33 ± 0.62	2.56 ± 0.63
Surgical score, 60	2.67 ± 0.62	2.44 ± 0.51
Surgical score, 90	2.80 ± 0.68	2.25 ± 0.58^*^
Total propofol dose (mg)	1127.4 ± 374.5	984.2 ± 304.1
Total remifentanil dose (mcg)	1221.2 ± 347.3	2192 ± 719.2^*^
VAS	4.19 ± 2.24	4.19 ± 2.35
PONV	0.11 ± 0.32	0.00 ± 0.00

*VAS* Visual analogue score, *PONV* Postoperative nausea and vomiting

^*^p < 0.05 compared to Group LR

## Discussion

The most important findings of this study are a significant decrease in the surgical score and cardiac output in the high-concentration remifentanil group compared to the low-concentration group. Non-invasive cardiac monitoring was used to confirm that this decrease is due to a reduction in cardiac output primarily attributed to the decrease in heart rate induced by remifentanil, rather than a decrease in stroke volume.

These findings have clinical significance as successful endoscopic sinus surgery relies on accurately identifying anatomical structures. Improved surgical conditions and enhanced visibility contribute to more precise surgical maneuvers and potentially reduce the risk of complications associated with increased bleeding, such as postoperative hemorrhage, cerebrospinal fluid leakage, and visual loss [7]. To mitigate these risks, the use of the reverse Trendelenburg position, topical decongestants, and vasoconstrictors has been suggested [8]. Additionally, controlled hypotension using general anesthesia has been employed to reduce bleeding during endoscopic sinus surgery [9]. The use of drugs such as vasodilators, sodium nitroprusside, or beta-blockers has also been proposed to lower blood pressure [10–12].

Total intravenous anesthesia using the intravenous anesthetic, propofol and the opioid, remifentanil is widely used to minimize intraoperative bleeding by reducing cardiac output without rapidly decreasing systemic vascular resistance [3]. This effect is likely due to the bradycardia-inducing effect of remifentanil, along with its blood pressure-lowering effect [13]. In this study, we confirmed a significant decrease in heart rate in the high-concentration group compared to the low-concentration group using a non-invasive cardiac monitoring device. Several reports suggest that the bradycardic effect of remifentanil is associated with its central vagotonic effect [14].

Although a significant decrease in cardiac output was observed in the high-concentration group, stroke volume did not show a significant difference between the two groups. Based on these results, the decrease in cardiac output in the high-concentration group appears to be more strongly related to the decrease in heart rate than to the decrease in stroke volume. Cheong et al. [15] reported no proportional decrease in myocardial contractility with increased remifentanil usage, nor a compensatory increase in myocardial contractility with a decrease in heart rate. Manola et al. [16] found that the blood pressure-lowering effect of remifentanil was due to the reduction of cardiac output rather than peripheral vasodilation.

In this study, cardiac output and stroke volume were measured using non-invasive cardiac monitoring (CSN-1901). The CSN-1901 monitoring system is based on pulse wave transit time and indirectly measures cardiac output and stroke volume through the R-wave of the electrocardiogram, the pulse waves of the pulse oximeter, and blood pressure. Traditionally, cardiac output has been measured invasively, such as with a pulmonary artery catheter, but the use of non-invasive methods is increasing while invasive methods are declining [17, 18]. Saugel et al. [17] compared the types and mechanisms of non-invasive cardiac monitoring with invasive measurement methods, and Jeon et al. [19] reported that using the CSN-1901 is helpful for estimating intravascular volume status.

It is important to acknowledge limitations of this study. Firstly, the evaluation of surgical conditions using the Boezaart grading scale, while widely used, is subjective and lacks standardization. Athanasiadis et al. [2] reported that most reported scores on the Boezaart grading scale were 2 or 3 points, making it difficult to detect subtle differences in the surgical field. Additionally, inter-rater reliability may vary, and reviewer bias is possible. Secondly, the study only measured cardiac output and stroke volume through non-invasive cardiac monitoring based on pulse wave transit time, which may not provide a comprehensive assessment compared to invasive methods like pulmonary artery catheterization or transesophageal echocardiography. Further studies comparing different methods of measuring cardiac output could provide more insights. Also, this study had a relatively small sample size, potentially limiting the ability to generalize the results to a broader population. Furthermore, the inclusion criteria restricted participation to patients classified as ASA I or II, excluding those with more severe health conditions, which could introduce bias. Additionally, the study was conducted in a single hospital, which may hinder the generalizability of the findings to hospitals in other regions or countries with different healthcare systems and care standards. Furthermore, long term surgical outcomes, length of hospital stay, and patient satisfaction may be targeted for future research.

In conclusion, the findings of this study support the use of high-concentration remifentanil during endoscopic sinus surgery to improve surgical conditions and reduce bleeding. The decrease in the surgical score and cardiac output in the high-concentration group indicates enhanced visibility and potential benefits for successful surgical outcomes. However, the subjective nature of the surgical grading scale and the limitations of non-invasive cardiac monitoring should be considered when interpreting the results. Additionally, a recent meta-analysis indicated that TIVA appears to have advantages in terms of visibility scores and blood loss during endoscopic sinus surgery compared to inhalational anesthesia. However, the inconsistency observed when stratifying the results based on the use of remifentanil and various inhaled anesthetics emphasizes the need for further investigation [20]. Further research exploring standardized evaluation methods and comparing different techniques for measuring cardiac output is warranted to strengthen the evidence base for optimizing anesthesia protocols in endoscopic sinus surgery.

## Data Availability

The datasets used and analyzed during the current study are available from the corresponding author on reasonable request.
